# Supplementary material for: Evaluating the motional timescales contributing to averaged anisotropic interactions in MAS solid-state NMR
Source: Magn Reson (Gott). 2024 Jun 11;5(1):69–86. doi: 10.5194/mr-5-69-2024 (PMC12082686; doi:10.5194/mr-5-69-2024)
Supplement: The supplement related to this article is available online at: https://doi.org/10.5194/mr-5-69-2024-supplement. [file mr-5-69-2024-supplement.pdf]

Supplement of Magn. Reson., 5, 69–86, 2024
https://doi.org/10.5194/mr-5-69-2024-supplement
© Author(s) 2024. CC BY 4.0 License.

Open Access
MAGNETIC RESONANCE

Supplement of

Evaluating the motional timescales contributing to averaged anisotropic interactions in MAS solid-state NMR

Kathrin Aebischer et al.

Correspondence to: Matthias Ernst (maer@ethz.ch)

The copyright of individual parts of the supplement might differ from the article licence.

Contents

S1 Summary of Simulation Parameters **1**

S2 Additional Figures **3**

S1 Summary of Simulation Parameters

Table S1. Summary of simulation parameters. In simulations of heteronuclear two-spin systems (CP, REDOR, wPARS and off-MAS) I spins correspond to protons and S spins correspond to ^{13}C. More than 538 powder orientations are only necessary for simulations of quadrupolar spectra. All simulation programs are freely available in the data repository.

Experiment	Initial Density Op. $\hat{\rho}_{\text{init.}}$	Detection Op. $\hat{D}$	No. of Crystallites M	Time Resolution Δt
CP	$\hat{I}_x$	$\sin\vartheta_1 \hat{S}_x + \cos\vartheta_1 \hat{S}_z$	10000	0.5 μs (20 kHz MAS)
				0.167 μs (60 kHz MAS)
				0.1 μs (100 kHz MAS)
REDOR	$\hat{S}_x$	$\hat{S}_x$	10000	0.25 μs (20 kHz MAS)
				0.042 μs (60 kHz MAS)
				0.05 μs (100 kHz MAS)
wPARS	$\hat{S}_x$	$\hat{S}_x$	10000	0.25 μs (20 kHz MAS)
				0.042 μs (60 kHz MAS)
				0.05 μs (100 kHz MAS)
off-MAS	$\hat{I}_x$	$\hat{I}_x$	538	0.5 μs (20 kHz MAS)
				0.167 μs (60 kHz MAS)
				0.1 μs (100 kHz MAS)
CSA recoupling	$\hat{I}_x$	$\hat{I}_x$	1154	0.556 μs (20 kHz MAS)
				0.185 μs (60 kHz MAS)
				0.111 μs (100 kHz MAS)
Quadrupolar spectra	$\hat{I}_x$	$\hat{I}^-$	10000	0.125 μs (20 kHz MAS)

Table S2. Summary of radio-frequency field strengths used for simulations of pulsed dipolar recoupling under MAS shown in the main text.

MAS	CP		REDOR		wPARS	
	$\nu_{1\mathrm{I}}$	$\nu_{1\mathrm{S}}$	$\nu_{1\mathrm{I}}$	$\nu_{1\mathrm{S}}$	$\nu_{1\mathrm{I}}$	$\nu_{1\mathrm{S}}$
20 kHz	93 kHz	73 kHz	100 kHz	100 kHz	100 kHz	100 kHz
60 kHz	37 kHz	23 kHz	100 kHz	100 kHz	300 kHz	300 kHz
100 kHz	63 kHz	37 kHz	1000 kHz	1000 kHz	500 kHz	500 kHz

S2 Additional Figures

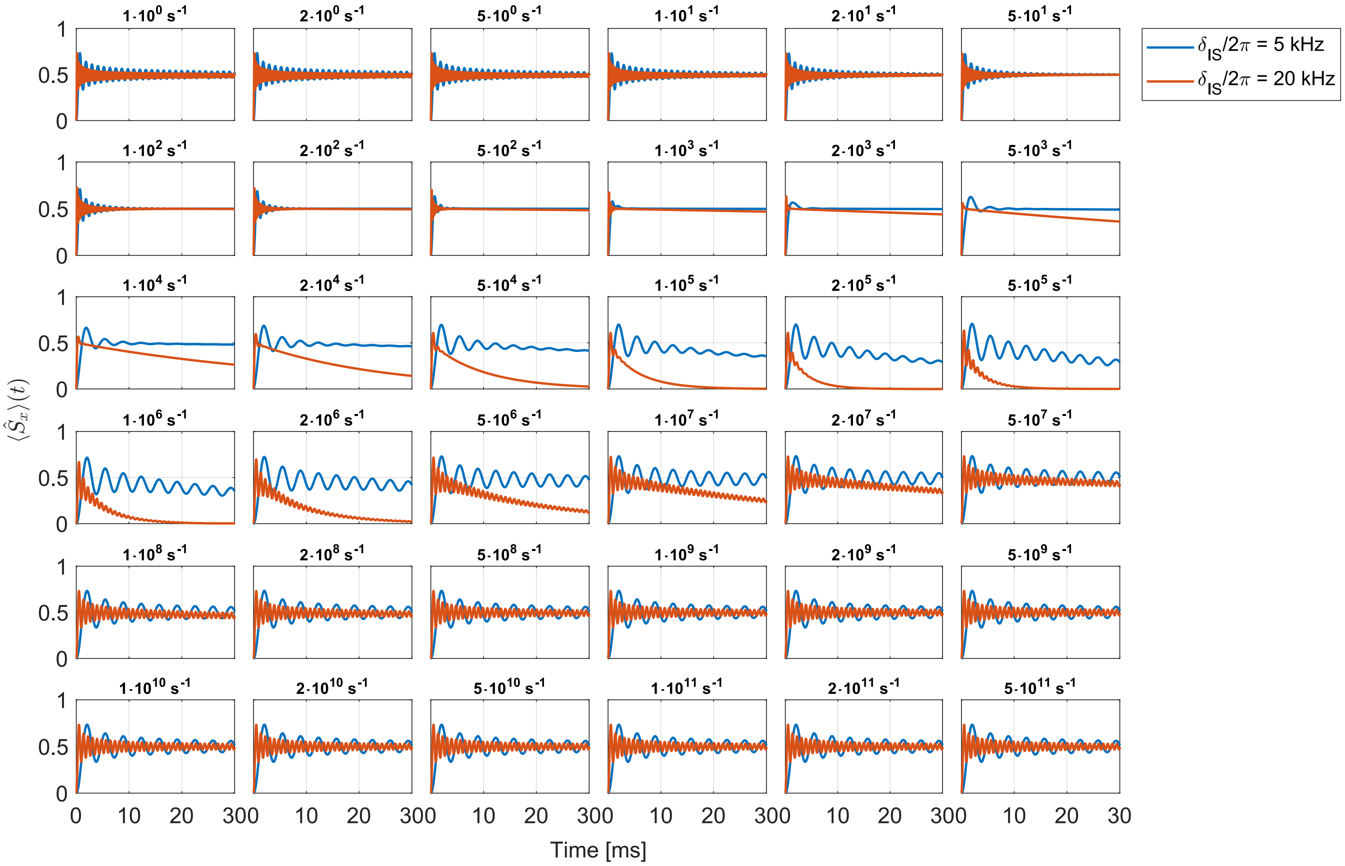

Figure S1. Simulated CP polarization transfer for a heteronuclear spin pair with different dipolar coupling strengths δ_{IS} for the $n = 1$ zero-quantum matching condition (see Table S2 for rf field strengths). Shown are the resulting recoupling curves for all simulated exchange-rate constants between $1\ \mathrm{s}^{-1}$ and $5 \cdot 10^{11}\ \mathrm{s}^{-1}$ for a MAS frequency of 20 kHz. In the intermediate exchange regime, signal decay due to rotating frame relaxation is observed. This mostly affects the recoupling curves for long contact times and only has a marginal effect on the initial build-up that is used in the χ^2 fit.

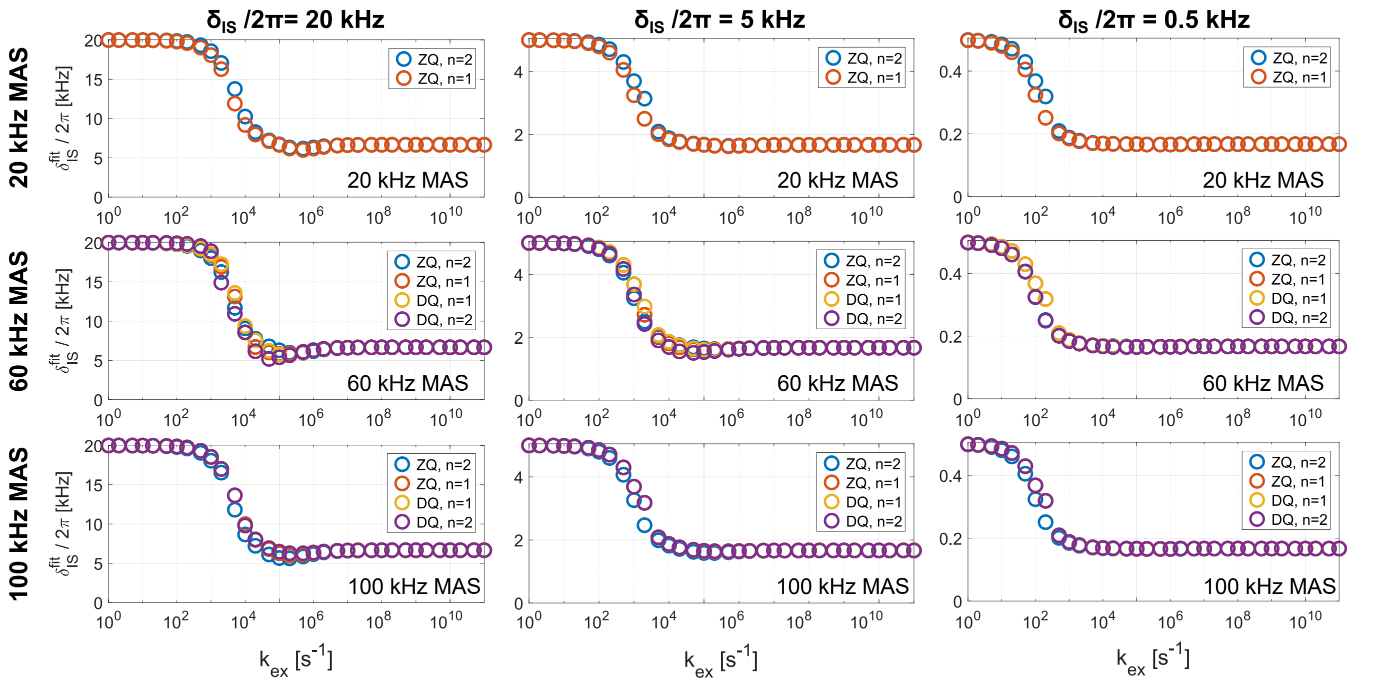

Figure S2. Fitted apparent δ_{IS}^{fit} for different CP matching conditions for 20, 60 and 100 kHz MAS (rows) and different dipolar coupling strengths (columns). For the zero-quantum (ZQ) matching conditions ($\nu_{1I} - \nu_{1S} = n\nu_r$) the following radio-frequency field amplitudes ($\nu_{1I} : \nu_{1S}$) were used: 113 kHz : 73 kHz ($n = 2$) and 93 kHz : 73 kHz ($n = 1$) for 20 kHz MAS; 157 kHz : 37 kHz ($n = 2$) and 97 kHz : 37 kHz ($n = 1$) for 60 kHz MAS; 277 kHz : 77 kHz ($n = 2$) and 177 kHz : 77 kHz ($n = 1$) for 100 kHz MAS. For the double-quantum (DQ) matching conditions ($\nu_{1I} + \nu_{1S} = n\nu_r$) the following radio-frequency field amplitudes ($\nu_{1I} : \nu_{1S}$) were used: 83 kHz : 37 kHz ($n = 2$) and 37 kHz : 23 kHz ($n = 1$) for 60 kHz MAS; 129 kHz : 71 kHz ($n = 2$) and 63 kHz : 37 kHz ($n = 1$) for 100 kHz MAS. The underlying molecular motion was modeled using a three-site jump process with an opening angle of $\theta = 70.5°$. The observed recoupling behaviour is very similar for all matching conditions. Differences are only observed in the intermediate exchange regime and can be attributed to the different rf field amplitudes that affect the rotating-frame relaxation during the contact time.

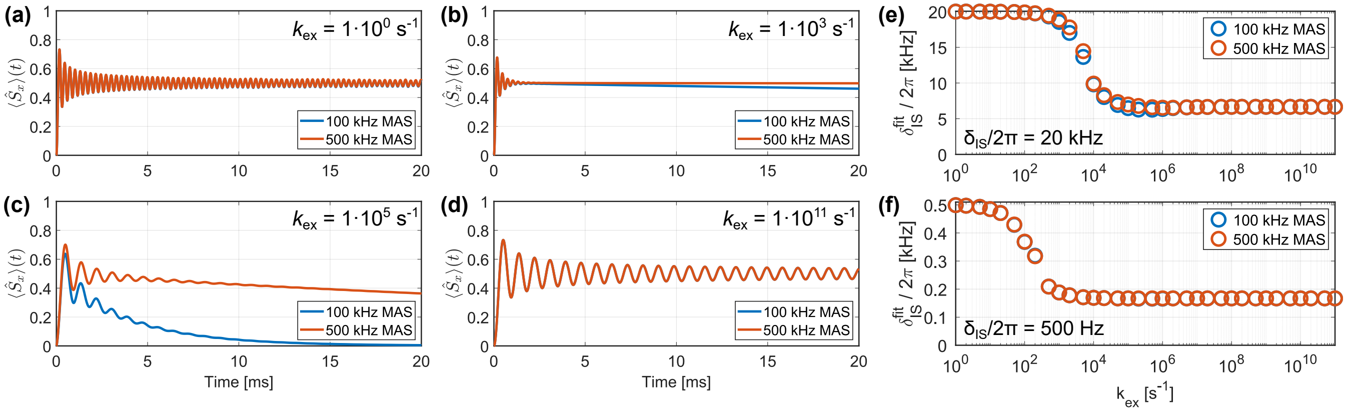

Figure S3. Simulated CP recoupling for 100 kHz and 500 kHz MAS for a heteronuclear spin pair. The underlying molecular motion was modeled as a three-site jump process with an opening angle of $\theta = 70.5°$. Radio-frequency field amplitudes ($\nu_{1\mathrm{I}} : \nu_{1\mathrm{S}}$) of: 177 kHz : 77 kHz (for 100 kHz MAS) and 283 kHz : 217 kHz (for 500 kHz MAS) were used. a-d) Comparison of recoupling curves for different exchange-rate constants for a dipolar coupling strength of $\delta_{\mathrm{IS}}/(2\pi) = 20$ kHz. In the limit of fast and slow exchange, the recoupling curves obtained for the two MAS frequencies are identical (a and d). In the intermediate exchange regime (b and c) loss of magnetization due to relaxation is significantly slower at 500 kHz. e-f) Fitted apparent $\delta_{\mathrm{IS}}^{\mathrm{fit}}$ as a function of the exchange-rate constant for dipolar coupling strengths of $\delta_{\mathrm{IS}}/(2\pi) = 20$ kHz (e) and $\delta_{\mathrm{IS}}/(2\pi) = 0.5$ kHz (f). No significant differences between the two spinning frequencies are observed.

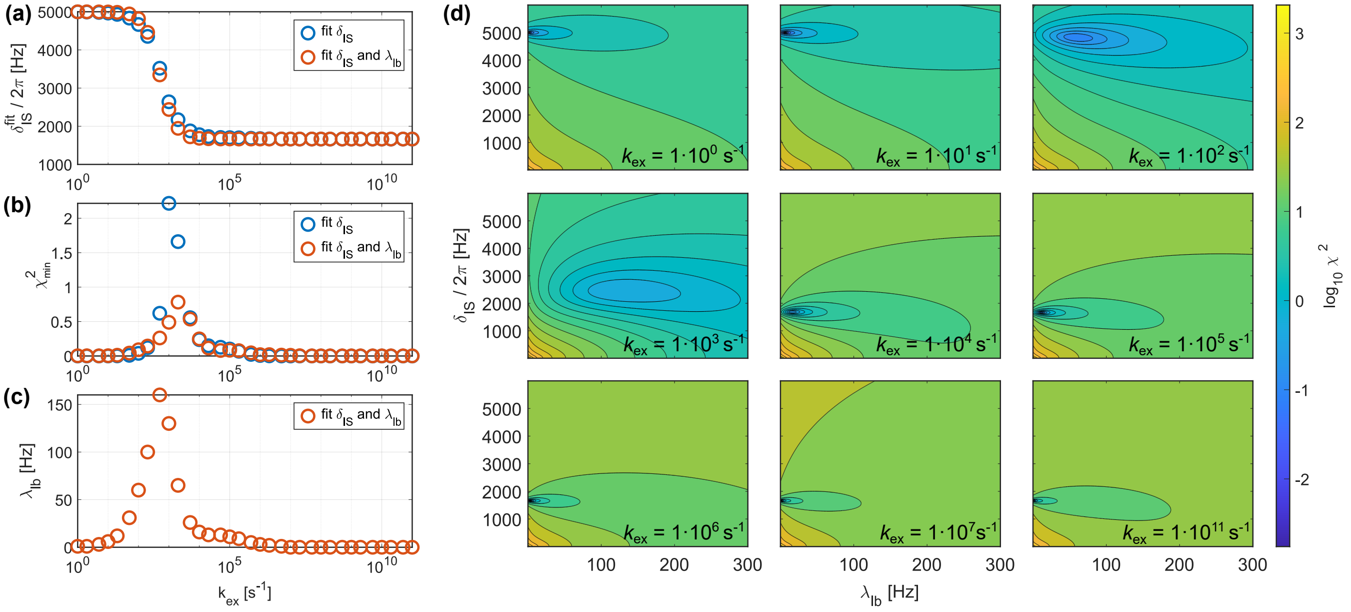

Figure S4. Comparison of χ^2-fitting routines for wPARS dipolar recoupling (20 kHz MAS, $\delta_{\mathrm{IS}}/(2\pi) = 5$ kHz, $\theta = 70.5°$): i) fit of only δ_{IS} (blue circles) and ii) fit of grid with δ_{IS} and an additional exponential broadening parameter λ_{lb} (applied as: $\exp(-\lambda_{\mathrm{lb}}\pi t)$ to the recoupling curve) to account for the decay of magnetization due to molecular motion (red circles). a) Resulting $\delta_{\mathrm{IS}}^{\mathrm{fit}}$ (and λ_{lb} in c) for the two fitting routines and the corresponding minimum χ^2_{min} value in b). d) Contour plots of χ^2 for the grid of λ_{lb} and δ_{IS} used for fitting. Significant broadening is observed for the intermediate exchange regime. However, including λ_{lb} in the fitting routine has a marginal influence on the obtained $\delta_{\mathrm{IS}}^{\mathrm{fit}}$ and only leads to a reduction of χ^2_{min}.

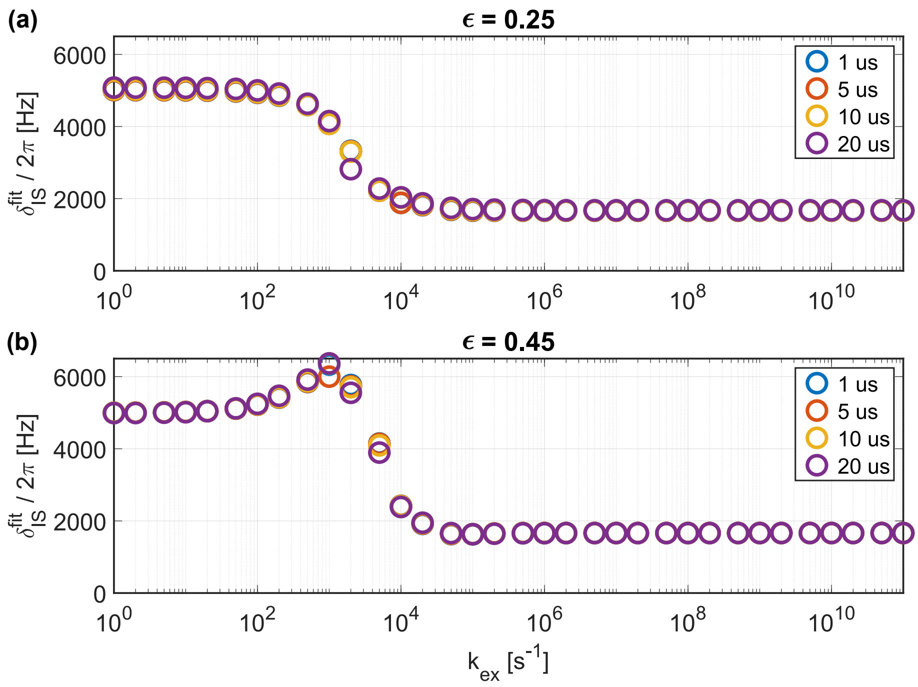

Figure S5. Comparison of the fitted apparent anisotropy of the dipolar coupling $\delta_{\mathrm{IS}}^{\mathrm{fit}}$ for REDOR recoupling with different rf field strengths (π-pulse lengths of 1, 5, 10 and 20 μs corresponding to rf field strengths between 25-500 kHz) for $\epsilon = 0.25$ (no pulse shifting, a) and $\epsilon = 0.45$ (π-pulses shifted to scale the heteronuclear dipolar coupling, b). Data is shown for a MAS frequency of 20 kHz and $\delta_{\mathrm{IS}}/(2\pi) = 5$ kHz and a three-site jump process with an opening angle of $\theta = 70.5°$. No significant differences are observed for the different pulse lengths.

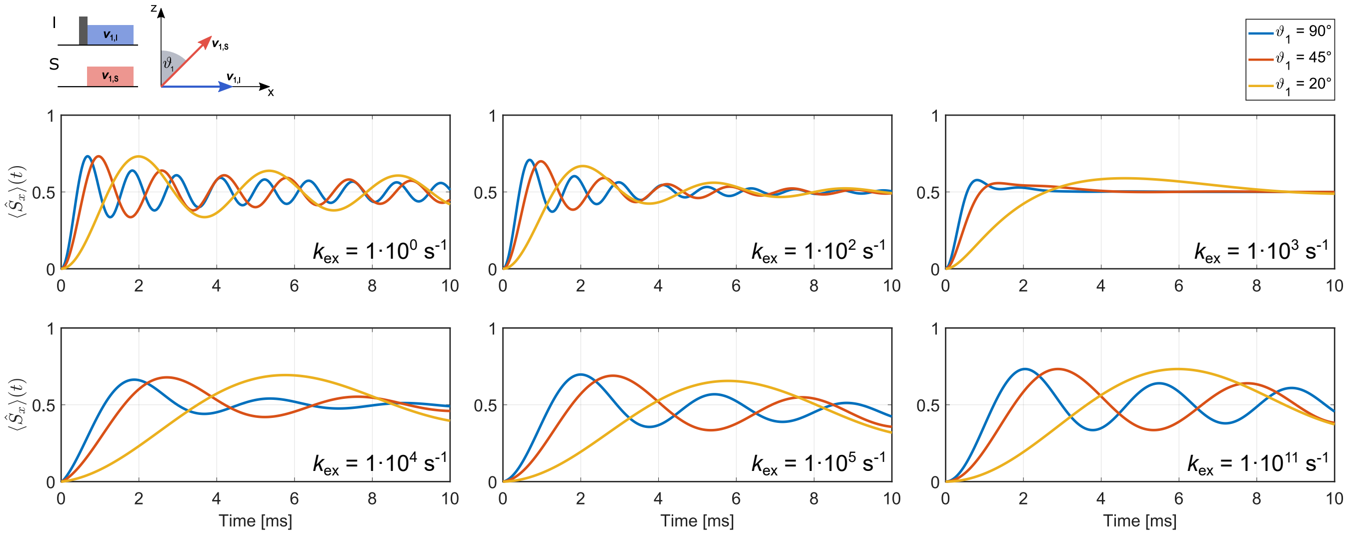

Figure S6. Effect of tilting the rf field on one of the two channels during the CP experiment on the observed recoupling behaviour in the presence of molecular motion. Data is shown for different tilt angles ϑ_1 for a MAS frequency of $20\,\text{kHz}$, $\delta_{\text{IS}}/(2\pi) = 5\,\text{kHz}$ and $\theta = 70.5°$. The rf field amplitudes were set to fulfill the $n = 1$ zero-quantum matching condition ($\nu_{1\text{I}} = 93$ kHz, $\nu_{1\text{S}} = 73\,\text{kHz}$).Tilting the rf field away from the transverse plane scales the heteronuclear dipolar coupling resulting in slower oscillations in the recoupling curve. The effects of molecular motion on the appearance of the recoupling curve are similar for all tilt angles.

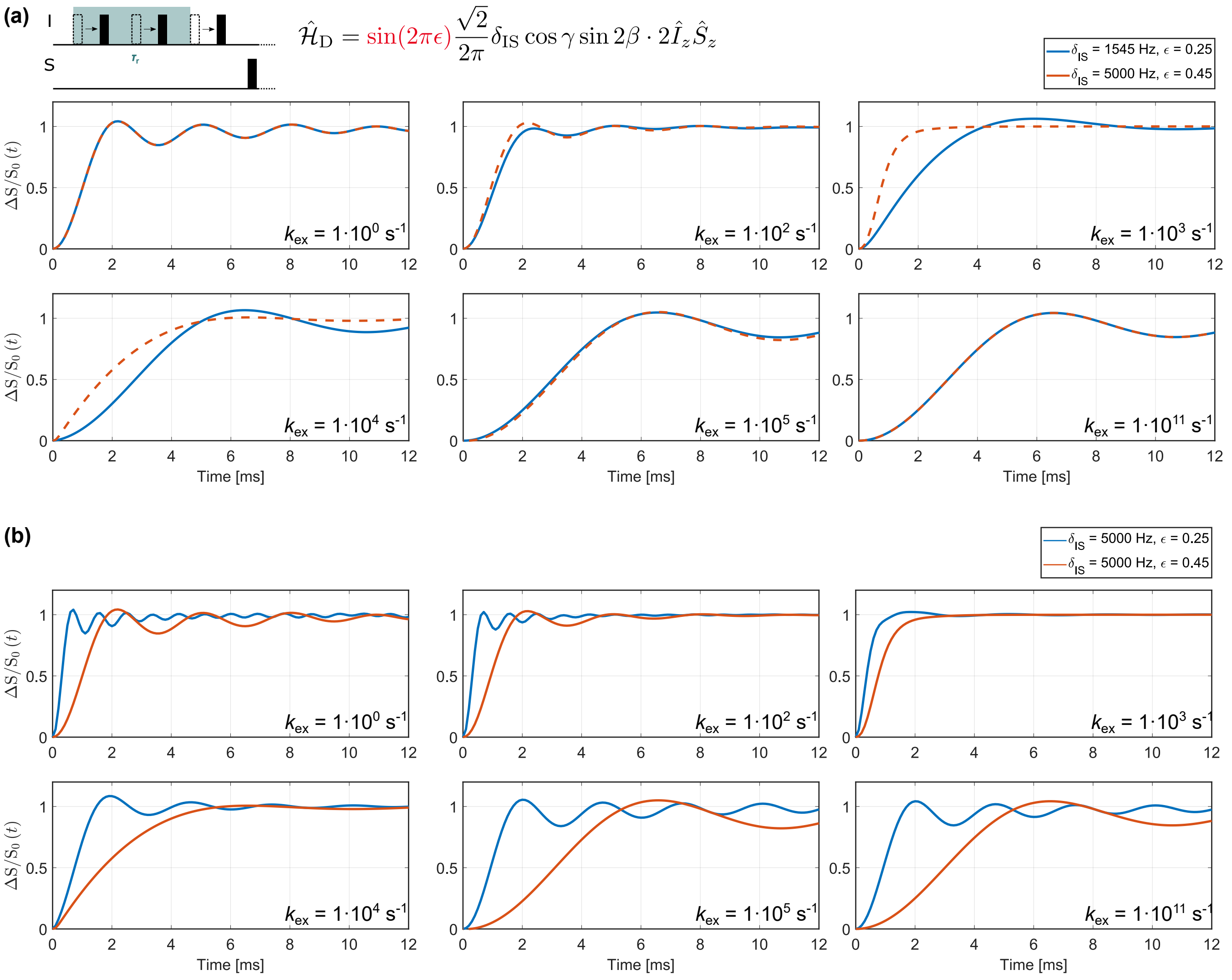

Figure S7. Effect of shifting the π-pulses in the REDOR sequence on the appearance of the resulting REDOR curve in dynamic systems. In this implementation of the REDOR sequence, the heteronuclear dipolar coupling can be scaled by shifting both π-pulses while keeping the pulse separation constant at $\tau_{\mathrm{r}}/2$. The extent of the pulse shift is characterized by the parameter $\epsilon = \frac{\tau_1 + 0.5\tau_{\mathrm{p}}}{\tau_{\mathrm{r}}} - 0.25$ (τ_1: beginning of the first π-pulse, τ_{p}: length of π-pulse, τ_{r}: rotor cycle), where $\epsilon = 0.25$ corresponds to the unshifted REDOR experiment and the dipolar coupling is scaled by $\sin(2\pi\epsilon)$. Data is shown for a MAS frequency of 20 kHz and an opening angle of the underlying three-site jump process of $\theta = 70.5°$. a) Comparison of simulated REDOR curves for $\delta_{\mathrm{IS}}/(2\pi) = 5000\,\mathrm{Hz}$ with shifted pulses ($\epsilon = 0.45$) and an unshifted REDOR experiment with $\delta_{\mathrm{IS}}/(2\pi) = 1545\,\mathrm{Hz}$ ($\epsilon = 0.25$). The shifting of the pulse position leads to a scaling of the anisotropy of the dipolar coupling given by $\delta_{\mathrm{IS}}/(2\pi) = 5000\,\mathrm{Hz} \cdot \sin(2\pi \cdot 0.45) \approx 1545\,\mathrm{Hz}$ which corresponds to the dipolar coupling used in the simulation of the unshifted REDOR experiment. In the limit of slow and fast exchange, identical REDOR curves are obtained. Shifting the π-pulses therefore simply leads to a scaling of the coupling in these exchange regimes. In the intermediate exchange regime on the other hand, shifting the position of the refocusing pulses changes the appearance of the REDOR curve significantly. For the shifted REDOR experiment, a rapid build-up of the curve without oscillations is observed that prevents the extraction of the coupling strength in this exchange regime. b) Comparison of the resulting REDOR curves for $\delta_{\mathrm{IS}}/(2\pi) = 5000\,\mathrm{Hz}$ for the unshifted ($\epsilon = 0.25$) and the shifted REDOR experiment ($\epsilon = 0.45$). Shifting the refocusing pulses leads to a scaling of the dipolar coupling and affects the appearance of the REDOR curve in the intermediate exchange regime.

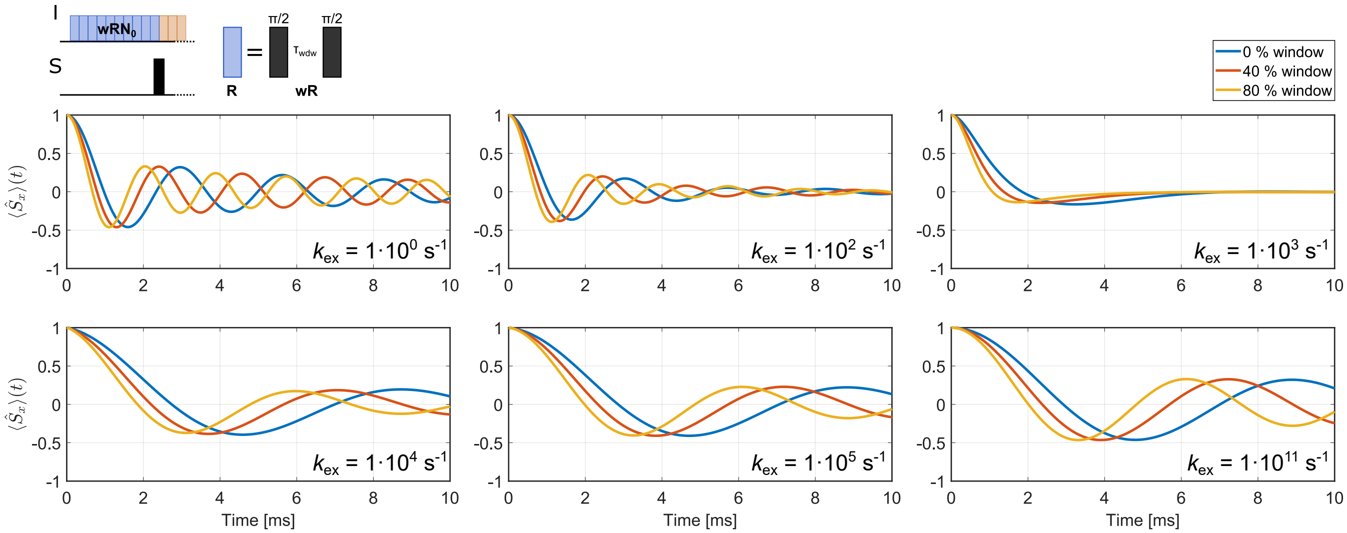

Figure S8. Effect of the introduction of a window in the basic R element of the wPARS sequence on the observed recoupling behaviour in dynamic systems. The basic R element consists of a π-pulse that can be separated into two $\frac{\pi}{2}$-pulses with a window without rf irradiation. This results in the scaling of the heteronuclear dipolar coupling and faster oscillations are observed for longer windows. The effects of molecular motion on the appearance of the recoupling curve are similar for all window fractions. Data is shown for a MAS frequency of 20 kHz, a dipolar coupling of $\delta_{\mathrm{IS}}/(2\pi) = 5$ kHz and $\theta = 70.5°$. The required radio-frequency field strength depends on the window fraction and increases with increasing window fraction.

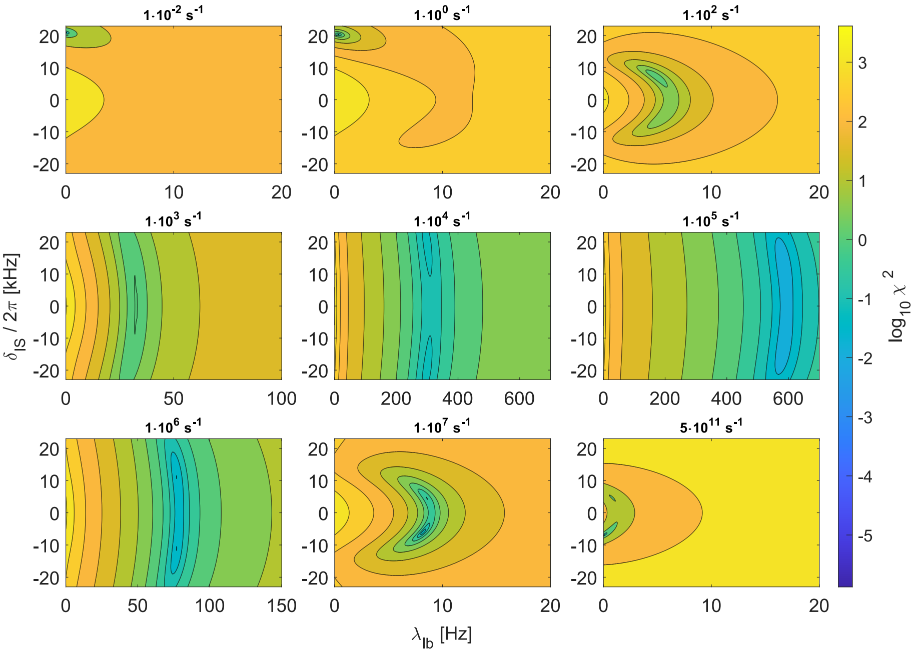

Figure S9. Contour plots of the χ^2 value for the two-dimensional λ_{lb} and δ_{IS} grid used for fitting the dephasing curves in off-magic-angle spinning simulations of a heteronuclear spin pair ($J =$ -90 Hz, $\delta_{\mathrm{IS}}/(2\pi) = 21$ kHz). Shown are results for an angle offset of $\Delta = 0.05°$ and a spinning frequency of 20 kHz for different exchange-rate constants for a three-site jump process with an opening angle of $\theta = 70.5°$. In the limit of slow ($k_{\mathrm{ex}} = 1 \cdot 10^{-2}\ \mathrm{s}^{-1}$) and fast ($k_{\mathrm{ex}} = 1 \cdot 10^{11}\ \mathrm{s}^{-1}$) exchange, the χ^2-minimum is located at the full and scaled interaction (order parameter of $S_{\mathrm{D}} = -\frac{1}{3}$). In the intermediate exchange regime (ca. $1 \cdot 10^{3}\ \mathrm{s}^{-1} < k_{\mathrm{ex}} < 1 \cdot 10^{6}\ \mathrm{s}^{-1}$) signal decay due to relaxation leads to an elongated χ^2-minimum prohibiting the extraction of a well-defined $\delta_{\mathrm{IS}}^{\mathrm{fit}}$. In the transition region (from full to scaled dipolar coupling, $k_{\mathrm{ex}} \approx 1 \cdot 10^{2}\ \mathrm{s}^{-1}$), the sign of the anisotropy of the dipolar coupling is not well defined.

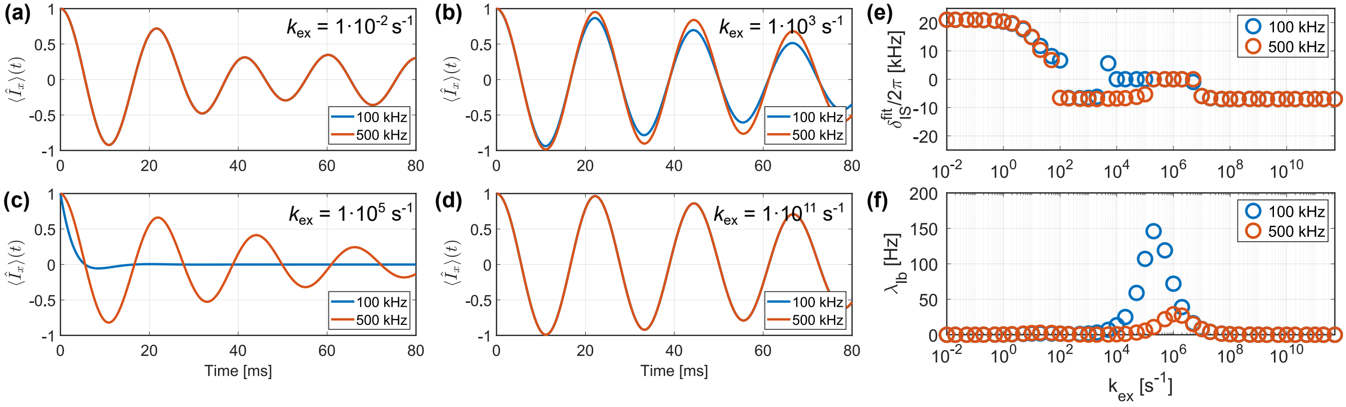

Figure S10. Simulated off-MAS dephasing curves for spinning frequencies of 100 kHz and 500 kHz for a heteronuclear spin pair with $\delta_{\mathrm{IS}}/(2\pi) = 20$ kHz for an angle offset of $\Delta = 0.05°$. The underlying molecular motion was modeled as a three-site jump process with an opening angle of $\theta = 70.5°$ corresponding to an order parameter of $S_{\mathrm{D}} = -\frac{1}{3}$. a-d) Comparison of dephasing curves for different exchange-rate constants. In the limit of fast and slow exchange, the dephasing curves obtained for the two spinning frequencies are identical (a and d). In the intermediate exchange regime (b and c) loss of magnetization due to relaxation is significantly slower at 500 kHz. e-f) Fitted apparent $\delta_{\mathrm{IS}}^{\mathrm{fit}}$ (e) and line broadening parameter λ_{lb} (f) as a function of the exchange-rate constant. For a spinning frequency of 500 kHz the range of exchange-rate constants where rapid relaxation (strong linebroadening) prevents the exctraction of $\delta_{\mathrm{IS}}^{\mathrm{fit}}$ is significantly narrower. Efficient relaxation is only observed for exchange-rate constants between ca. $1 \cdot 10^5$ s^{-1} - $1 \cdot 10^7$ s^{-1}. Therefore, the full transition from the full to the scaled coupling can be characterized.

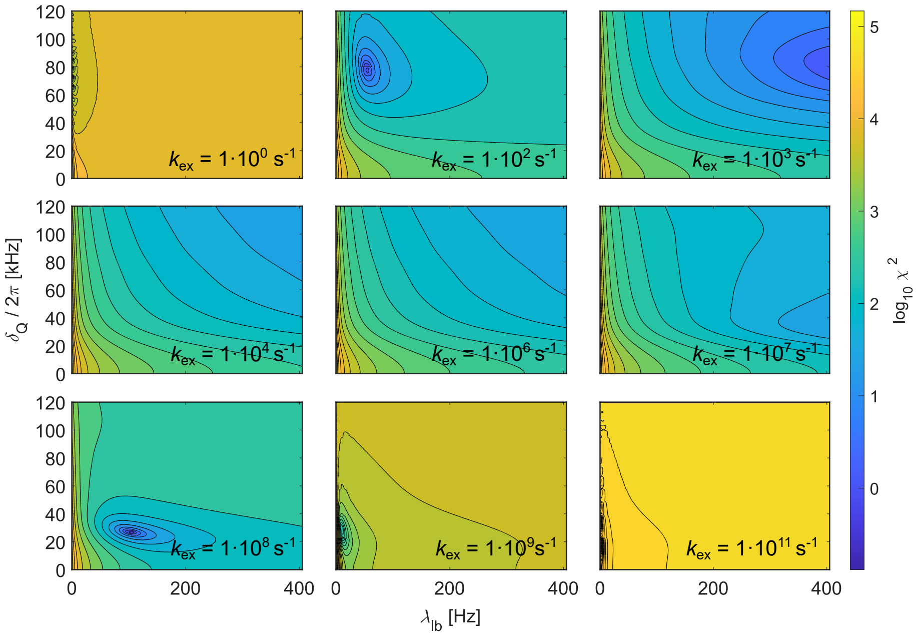

Figure S11. Contour plots of χ^2 for the grid of λ_{lb} and δ_{Q} used for fitting of FIDs of ^{2}H under MAS with a spinning frequency of 20 kHz for different exchange-rates. Prior to χ^2-fitting, a frequency shift is applied to the FID to ensure that the central peak in the spectrum corresponds to a shift of 0 Hz. The anisotropy of the quadrupolar coupling tensor was set to $\delta_{\mathrm{Q}}/(2\pi) = 80$ kHz and the opening angle of the exchange process was assumed to be $\theta = 70.5°$. In the intermediate exchange regime, the line broadening observed in the simulations exceeds the grid considered for the fitting. For fast exchange, small differences in the position of the central transition due to second-order quadrupolar contributions lead to large χ^2 values.

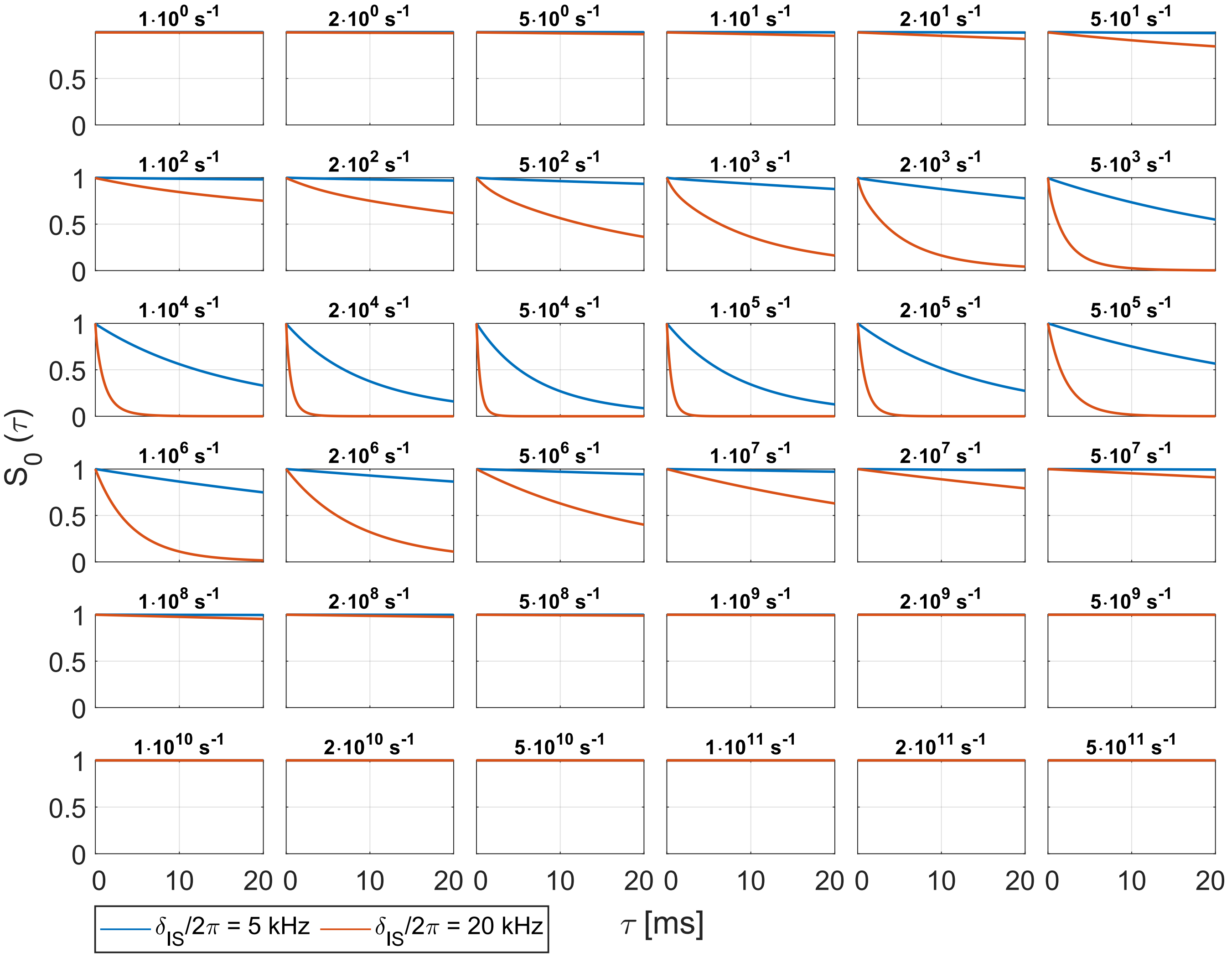

Figure S12. Decay of the simulated S_0 signal acquired during the REDOR reference experiment (without refocusing pulses on the I channel) for different exchange-rate constants. Data is shown for a MAS frequency of 20 kHz and two different dipolar coupling strengths ($\delta_{\mathrm{IS}}/(2\pi) =$ 20 and 5 kHz). The opening angle of the exchange process was assumed to be $\theta = 70.5°$. This reference experiment is used to account for the signal loss due to T_2 relaxation. Relaxation is observed for motion on intermediate time scales. A qualitative measure for the signal decay is the time required for the signal to decay below a value of $1/e$. The extracted $R_{1/e} = \frac{1}{T_{1/e}}$ time is shown in Fig. 5 in the main text as a function of the exchange-rate constant for different coupling strengths and MAS frequencies.
